# Relative Stability of Regional Facial and Ocular Temperature Measurements in Healthy Individuals

**DOI:** 10.1167/tvst.11.12.15

**Published:** 2022-12-29

**Authors:** Eleonora Micheletti, Nevin W. El-Nimri, Robert N. Weinreb, John H. K. Liu

**Affiliations:** 1 Viterbi Family Department of Ophthalmology, Shiley Eye Institute, University of California, San Diego, La Jolla, CA, USA

**Keywords:** cornea, forehead, infrared thermography, inner canthus, temperature

## Abstract

**Purpose:**

Non-contact measurement of facial temperature using infrared thermography has been used for mass screening of body temperature during a pandemic. We investigated the relative stability of temperature measurement in different facial regions of healthy individuals.

**Methods:**

Twenty healthy subjects underwent two experiments. In the first experiment, subjects washed their faces with a 20°C wet towel for 1 minute. Temperature changes compared to baseline in the forehead, cornea, inner canthus, and outer canthus were determined using an infrared camera for 10 minutes. In the second experiment, lubricating eye drops at 20°C were instilled over one eye. Temperature changes in the same regions of interest were monitored for 5 minutes.

**Results:**

Baseline temperatures before face washing in the forehead and cornea, inner canthus, and outer canthus of the right eye were 33.4°C ± 0.8°C (mean ± SD), 33.3°C ± 0.8°C, 34.3°C ± 0.7°C, and 32.8°C ± 0.7°C, respectively. Reductions in temperature due to face washing were most significant for the forehead and least significant for the cornea. One minute after face washing, the corresponding changes were −2.8°C ± 0.6°C, −0.3°C ± 0.6°C, −0.6°C ± 0.7°C, and −0.9°C ± 0.7°C for the forehead, cornea, inner canthus, and outer canthus, respectively. After administering the eye drops, no significant temperature changes were observed.

**Conclusions:**

When facial temperature was exogenously cooled, the cornea had the most stable temperature readings.

**Translational Relevance:**

When using infrared thermography to screen facial temperature, the measurement of corneal temperature is probably a better representative if the stability of temperature readings is critical.

## Introduction

Measurement of body temperature is a common method for detecting infectious diseases and has been used with flu strains such as H1N1 and severe acute respiratory syndrome. With the advent of the COVID-19 pandemic, in particular, there has been an increasing need for a simple and safe method to screen elevated body temperature. Non-contact facial temperature measurement using infrared thermography is the most frequently used technique to screen for access to hospitals, airports, and other restricted areas. There are questions associated with this approach, however, particularly with regard to environmental temperature, humidity, noise, direct sunlight, sweaty face, various social and psychological factors, and technical variations among infrared cameras, all of which can affect the reliability of measurement.^1^^–^^9^ For instance, a forehead temperature measurement underestimates the body temperature at building entrances with a cooler outdoor temperature.^7^^,^^9^ The stability of temperature measurement is an issue.

Previous investigations have attempted to identify regions of the face that are better correlated with body temperature when using infrared thermography.^10^^,^^11^ Reports have indicated that the inner canthus of the eye most closely reflects body temperature.^10^^,^^11^ Although the reliability of temperature measurement in this facial region as an indicator of body temperature has been questioned,^12^^,^^13^ the stability of temperature measurement of the inner canthus has not been studied. In the current study, we evaluated the relative stability of regional facial temperatures measured by infrared thermography in a cohort of healthy subjects whose faces responded to acute, exogenous cooler temperatures. Regions of interest were the forehead, cornea, inner canthus, and outer canthus. These facial regions are easily imageable even when a person wears a protective face mask during a pandemic.

## Methods

The study enrolled 20 healthy individuals who were 18 to 80 years old. Exclusion criteria were a history of temperature-dependent skin disorders, such as Raynaud's syndrome, cold panniculitis, or cryoglobulinemia; a history of ocular injury or ocular surgery; a diagnosis of any eye disease; the presence of moderate to severe dry eye; allergic keratoconjunctivitis or infectious keratitis; and contact lens wear. The subjects were informed about the experimental procedures. An informed consent form was signed by each subject prior to enrollment. The study protocol was approved by the Institutional Review Board of Human Research Protections Program at the University of California, San Diego, and it followed the tenets of the Declaration of Helsinki.

Experiments were performed indoors between 2 PM and 5 PM. The temperature and humidity of the room were recorded at each study visit. Infrared images were obtained using the infrared camera of a small, portable FLIR ONE Pro device (Teledyne FLIR, Santa Barbara, CA) that captured naturally emitted radiation in the 8- to 14-µm thermo-imaging wavelength.^14^ This infrared camera had a focal plane array size of 160 × 120. A temperature range of −20°C to 120°C was selected. Visible light images of 1440 × 1080 pixels were also obtained simultaneously by the digital camera of the FLIR ONE Pro device. The device was attached to an Apple iPad (8th generation; Apple Inc., Cupertino, CA) held by a desk stand to store the infrared and visible light images. A head and chin rest was used to position the head for imaging the whole face with both eyes open at a fixed distance of 15 cm from the infrared camera when data were collected.

The study consisted of two experiments performed in the same subjects. The first experiment monitored the temperature changes compared to baseline for the forehead, cornea, inner canthus, and outer canthus after face washing. Subjects were instructed to dab their entire faces with a wet towel at a controlled temperature of 20°C for 1 minute. Infrared images were collected immediately before (baseline) and after the face washing at preselected time points of 0.5, 1, 1.5, 2, 3, 4, 5, 6, 8, and 10 minutes.

**Figure 1.**
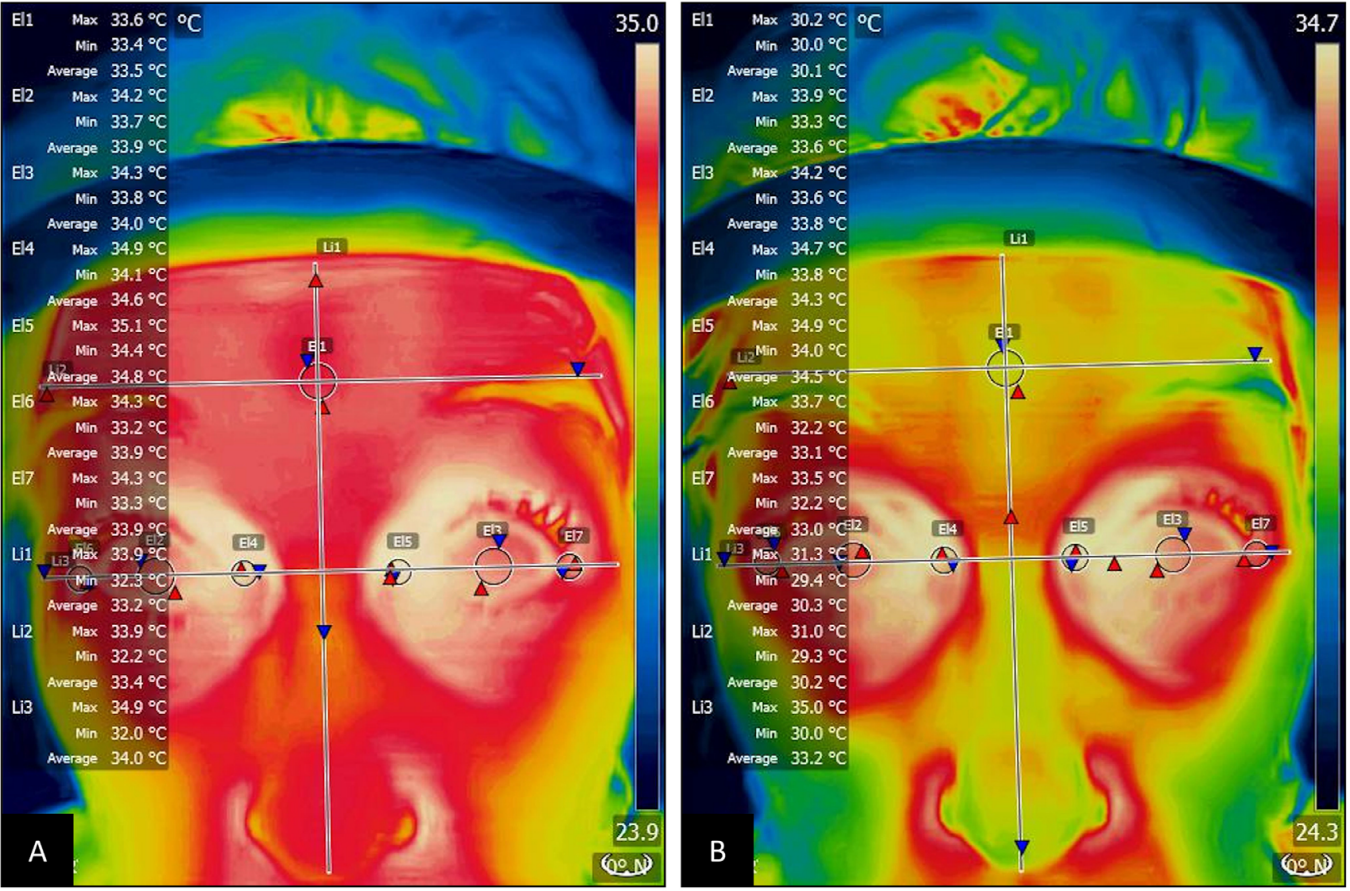
Temperature determinations of the forehead, central cornea, inner canthus, and outer canthus: (A) baseline, and (B) after face washing. Maximum (*red triangles*), minimum (*blue triangles*), and average temperatures in each region of interest—circle/ellipse (El) or line (Li)—were obtained automatically.

The infrared and visible light images were transferred offline to a computer and processed using FLIR Tools Thermal Analysis and Reporting, version 6.4, which automatically resized the infrared images to 640 × 480 pixels. Regions of interest were manually defined on the infrared image. The FLIR MSX Alignment function with visible light imaging was used to assist the process, particularly for the regions of inner and outer canthi that were centered at the junctions formed by the upper and lower eyelids. The average temperatures in the regions of interest were obtained automatically. Demarcations of the regions of interest are shown in Figure 1. To determine the forehead temperature, a perpendicular line was drawn to divide the nose into two symmetrical sections, and a horizontal line was drawn to connect the eyebrows. One circle with a diameter of 26 to 27 pixels (approximately 6.5 mm) was drawn around the crossing point between the two lines. Another horizontal line connecting the inner and outer canthi of each eye was drawn. Four circles with diameters 19 to 20 pixels (approximately 5 mm) were demarcated for the regions of canthus that contained sections of conjunctiva, upper eyelid, and lower eyelid. Similarly, two circles with diameters of 26 to 27 pixels were drawn around the central corneas. The selected sizes of forehead and central cornea could be defined in all infrared images without interference by hair strands, eyelids, or eyelashes.

During the face washing in the first experiment, the subjects’ eyes were probably closed and received little direct impact of the experiment. Therefore, the second experiment was performed to investigate the regional temperatures after challenging the ocular surface temperature with 20°C lubricating eye drops. Normal ocular surface temperature was expected in the range of 33°C to 36°C.^15^ After the regional facial temperatures in the first experiment had returned to normal (15–20 minutes), the examiner administered one drop of Refresh Plus lubricant eye drops (Allergan, Irvine, CA), which had been stored in a 20°C water bath, to one randomized eye of each subject. One drop of applied lubricant was about 41 µL, significantly larger than the normal tear volume of several microliters on the ocular surface.^16^ Infrared and visible light images were collected immediately before (baseline) and after administration of the eye drops at preselected time points of every half minute for 5 minutes. The regional temperatures were assessed similarly as in the first experiment.

Data of average temperature in the regions of interest were analyzed. Repeated-measures analysis of variance (ANOVA) was used to compare the temporal data in the forehead, cornea, inner canthus, and outer canthus in each experiment. Post hoc Bonferroni *t*-tests were used for comparisons between each post-treatment time point and the baseline. In addition, paired *t*-tests were used to compare the treatment effects between the two experiments in the 20 eyes that received the eye drop (nine right eyes and 11 left eyes). The criterion for statistical significance was *P* < 0.05.

## Results

**Figure 2.**
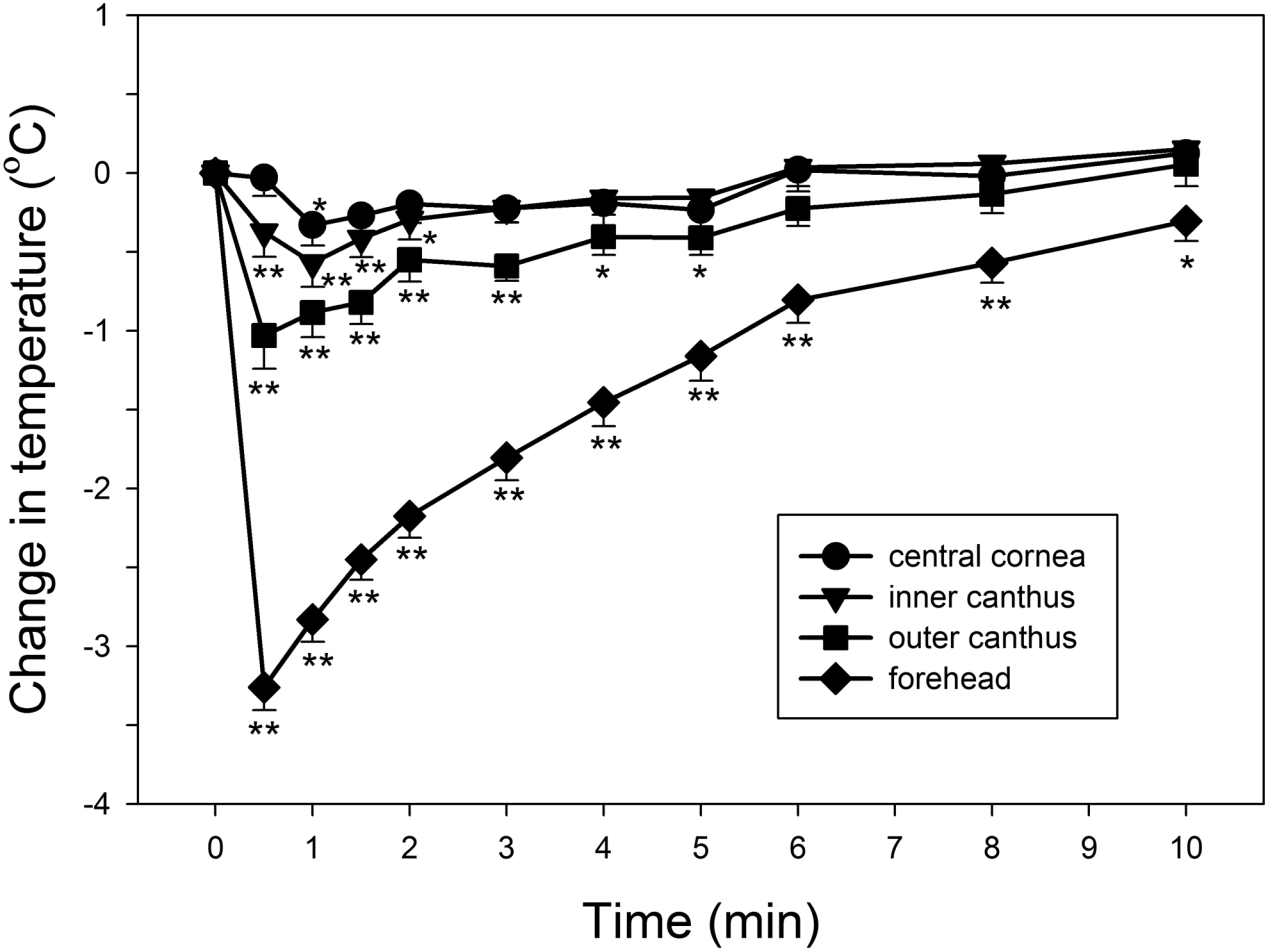
Temperature changes in the regions of interest after face washing. *P < 0.05 and **P < 0.01 compared to the baseline (repeated-measures ANOVA with post hoc Bonferroni *t*-test; *N* = 20). *Error bars* represent standard error of the mean.

The demographic characteristics of the 20 study subjects are presented in the Table. The examination room temperature was 21.1°C ± 1.7°C (mean ± SD) and humidity was 60.9% ± 1.9%. Baseline temperatures for the forehead and the cornea, inner canthus, and outer canthus in the right eye were 33.4°C ± 0.8°C, 33.3°C ± 0.8°C, 34.3°C ± 0.7°C and 32.8°C ± 0.7°C, respectively, in the first experiment. Face washing caused various temperature changes in the regions of interest (Fig. 2). Forehead temperatures decreased significantly for 10 minutes. The decrease was over 2°C for 2 minutes and gradually returned to the baseline. Compared to the forehead temperature reduction, temperature reductions in the ocular regions were all relatively shorter and smaller, not changing by more than 1°C. Corneal temperature showed the least temperature change of no more than 0.3°C compared to the baseline. At 1 minute after the face washing, when only the change of corneal temperature was statistically significant, corresponding temperature changes were −2.8°C ± 0.6°C, −0.3°C ± 0.6°C, −0.6°C ± 0.7°C, and −0.9°C ± 0.7°C in the forehead, cornea, inner canthus, and outer canthus. Similar patterns of changes in ocular temperatures compared to the forehead temperature appeared in the left eyes.

**Table.** Demographics of the 20 Study Subjects

Demographic	
Age (y), mean ± SD	42.4 ± 14.3
Gender (female/male), *n*	8/12
Race, *n*	
Caucasian	10
Hispanic	5
Asian	5
Height (cm), mean ± SD	171.2 ± 9.7
Weight (kg), mean ± SD	75.8 ± 22.6
Body mass index, mean ± SD	25.8 ± 7.0

**Figure 3.**
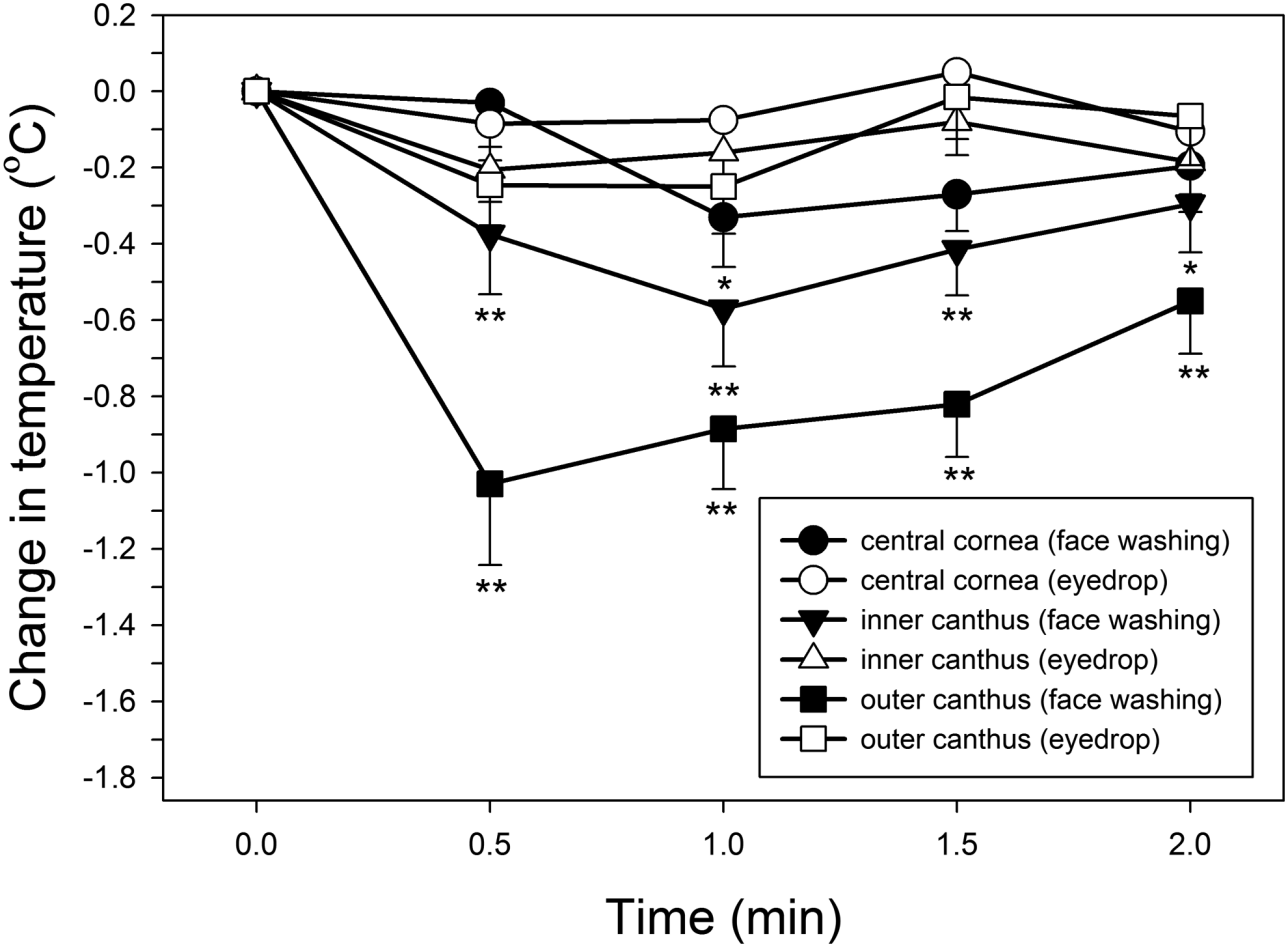
Ocular temperature changes after eye drop administration versus face washing. *P < 0.05 and **P < 0.01 (paired *t*-test between the two experiments; *N* = 20). *Error bars* represent standard error of the mean.

Temperature changes after the administration of eye drops in the second experiment were in the ranges of −0.1°C to 0.1°C for the forehead, −0.2°C to 0.1°C for the cornea, −0.2°C to 0°C for the inner canthus, and −0.3°C to 0.1°C for the outer canthus during the 5-minute monitoring period. No change was statistically significant (*P* > 0.05 for all). Figure 3 compares the temperature changes between the two experiments in the ocular regions during the first 2 minutes post-treatment. Temperature changes after the administration of eye drops were in general smaller than the temperature changes after the face washing.

## Discussion

Face washing with 20°C wet towel caused a significant and prolonged temperature reduction in the forehead. Temperature reductions in the inner canthus and outer canthus were smaller and shorter. The impact of face washing on corneal temperature was the least. With a direct challenge of 20°C eye drops applied to the ocular surface, the superior stability of corneal temperature was verified. The baseline regional facial temperatures in the current study (32.8°C–34.3°C) were all within a few degrees less than the anticipated normal body temperature (approximately 37°C). The temperature of the inner canthus was closer to the normal body temperature than other facial regions, as reported previously by others.^10^^,^^11^ However, the temperature of the inner canthus changed more than that of the cornea with face washing in the current study.

Corneal temperature is correlated with core body temperature, although the relationship may not be linear.^15^^,^^17^ Corneal temperature is also correlated with the diurnal rhythm of body temperature,^18^ which was verified in a separate group of 16 healthy individuals using a similar method of image analysis described in the current report (see Supplementary Table S1). Physiologically, temperature in the avascular cornea is regulated by heat exchanges with the external environment, eyelids and associated facial skin, aqueous humor, and tears.^19^ The latter two factors are uniquely significant for the cornea among the four facial regions studied. As the corneal surface represents the anterior boundary of the heat gradient across the eyeball, ocular surface temperature is regulated to some degree by the flow of aqueous humor.^15^^,^^20^ Aqueous humor has a slow 1% turnover rate per minute, approximately 2.75 µL.^21^ Therefore, compared to other facial regions, corneal temperature can better sustain a modest acute temperature change in the external environment and associated temperature changes in facial blood circulation.^22^

Tears also play a role in the regulation of corneal temperature. Blinking coats the ocular surface with warmer fresh tears and helps in maintaining the tear volume on the ocular surface.^15^^,^^23^ The central corneal temperature is approximately 0.5°C less than the temperature of the limbus, and normal tear flow rate is approximately 1.2 µL/min.^16^^,^^23^^,^^24^ It was reported that tear secretion may increase by stimulating the cold thermoreceptors in central cornea.^25^^,^^26^ When tears on the ocular surface were replaced with 20°C lubricant in the current study, the treatment had little impact on the central corneal temperature starting at half a minute after instillation. These observations suggest that the cornea can maintain its temperature when directly encountering a modest acutely cooler exogenous temperature. Thus, when responding to temperature fluctuations in the environment, corneal temperature is likely more stable than the temperature of the inner canthus, outer canthus, and forehead due to the physiological mechanisms in the regulation of corneal temperature.

Many environmental factors including ambient temperature, humidity, noise, and background infrared intensity, as well as physiological and psychological factors, that may or may not be associated with environmental variables can affect the accuracy of temperature determined by an infrared camera, in comparison with the subject's core body temperature.^1^^–^^6^^,^^8^^,^^9^^,^^11^^–^^13^ Instrumental factors of the infrared camera used also affect the accuracy.^9^^,^^14^ In the current study, all of these factors may affect the accuracy of temperature readings for the four facial regions of interest. We assumed that the regional impacts due to the confounding environmental, physiological, psychological, and instrumental factors unrelated to the two experimental treatments were not significantly different. Our focus was the stability of temperature measurements compared to the pretreatment baselines, not the temperature accuracy. The relative stability was observed when regional data from the same infrared images were analyzed.

Infrared thermography of the forehead and, to some extent, the inner canthus has been used as a first-line screening tool during the COVID-19 pandemic.^8^ For those infrared cameras already in use for mass screening of elevated body temperature, the measurement of corneal temperature appears to be a better alternative if the stability of temperature readings is critical. For example, corneal temperature may represent indoor facial temperature better than the forehead temperature when a person enters the building from outdoors with a cooler temperature.^7^^,^^9^ Corneal temperature may also be a better index of body temperature than the forehead temperature when the person has encountered abnormal temperatures associated with professional work or training in, for example, the food industry, laboratory research, athletic competition, or military service.^2^^,^^9^

Although small, inexpensive infrared cameras attached to smartphones and similar devices are not yet validated for the detection of fever, these devices are widely available for personal use, including taking facial photographs of oneself. Although the spread of infection during a pandemic is a concern, infrared thermography using photographic self-portraits can provide unlimited assessments of facial temperature with no need to include an additional operator. The platform demonstrated in the current study also has the potential to provide unbiased records of facial and ocular temperatures by telecommunication when a person is in isolation or is remote.

The current study has several limitations. First, core body temperatures were not measured. The relationships between corneal temperature and core body temperature in healthy individuals and in patients with fever, with and without antipyretic treatments, require additional studies with sufficient numbers of subjects so an algorithm for temperature conversion to core body temperature and threshold temperature for detecting fever can be developed with accuracy. Second, the applicability of results from the current study to different patient groups with ocular surface abnormalities, dry eye, ocular inflammation, or other ocular diseases, as well as individuals wearing contact lenses, must be clarified.^15^^,^^23^^,^^24^^,^^27^^–^^31^ Finally, the current study did not control the timing of the photographs after blinking and did not compare different shapes and sizes of central corneal areas for temperature calculations.^30^ The influences of these parameters on the stability of temperature measurement are unknown. All of these limitations must be addressed before considering the measurement of corneal temperature using infrared thermography as a more reliable indicator of body temperature for large-scale temperature screening.

In conclusion, the current study demonstrated that corneal temperature was the most stable measurement of facial temperature when challenged with a modest cooler exogenous temperature. In comparison, the forehead, inner canthus, and outer canthus temperatures were more affected.

## Supplementary Material

Supplement 1
