# Supplementary material for: Relative Stability of Regional Facial and Ocular Temperature Measurements in Healthy Individuals
Source: Transl Vis Sci Technol. 2022 Dec 29;11(12):15. doi: 10.1167/tvst.11.12.15 (PMC9804027; doi:10.1167/tvst.11.12.15)
Supplement: Supplement 1 [file tvst-11-12-15_s001.pdf]

Table S1. Morning-afternoon changes in the axillary temperature and the corneal temperature of right eye.

subject	morning temperature (8-11 AM)		afternoon temperature (2-5 PM)		Difference (afternoon minus morning)	
	axillary	corneal	axillary	corneal	axillary	corneal
1	35.6	34.2	36.2	34.7	0.6	0.5
2	35.9	31.5	36.5	33.0	0.6	1.5
3	35.4	31.2	36.5	33.9	1.1	2.7
4	35.9	33.5	36.5	34.9	0.6	1.4
5	35.2	33.4	35.9	33.7	0.7	0.3
6	34.8	33.2	35.6	33.3	0.8	0.1
7	36.2	32.5	36.5	33.3	0.3	0.8
8	35.5	30.7	36.7	33.0	1.2	2.3
9	36.0	34.1	36.2	33.2	0.2	-0.9
10	35.6	31.8	36.7	33.5	1.1	1.7
11	34.8	34.2	36.2	35.3	1.4	1.1
12	35.2	33.4	36.4	35.5	1.2	2.1
13	35.6	32.6	36.2	31.7	0.6	-0.9
14	35.4	33.2	36.1	34.2	0.7	1.0
15	35.5	32.9	36.2	32.5	0.7	-0.4
16	35.5	33.1	36.0	33.4	0.5	0.3
Mean	35.51	32.84	36.28	33.69	0.77	0.85
SD	0.39	1.06	0.30	1.02	0.34	1.08

Morning-afternoon temperature difference was statistically significant ($P < 0.01$, paired t-test) in the axillary temperature as well as in the corneal temperature. The changes in the axillary and corneal temperatures were correlated ($P < 0.01$, $r = 0.656$, linear regression). There was no significant correlation ($P > 0.05$) between the axillary temperature and the corneal temperature when analyzing the morning data, the afternoon data, or combined morning and afternoon data.
